# Adolescent-parent interactions and attitudes around screen time and sugary drink consumption: a qualitative study

**DOI:** 10.1186/1479-5868-6-61

**Published:** 2009-09-09

**Authors:** Libby A Hattersley, Vanessa A Shrewsbury, Lesley A King, Sarah A Howlett, Louise L Hardy, Louise A Baur

**Affiliations:** 1 Physical Activity Nutrition Obesity Research Group, University of Sydney, Sydney, Australia; 2 Discipline of Paediatrics and Child Health, University of Sydney, Sydney, Australia

## Abstract

**Background:**

Little is known about how adolescents and their parents interact and talk about some of the key lifestyle behaviors that are associated with overweight and obesity, such as screen time (ST) and sugary drink (SD) consumption. This qualitative study aimed to explore adolescents' and parents' perceptions, attitudes, and interactions in regards to these topics.

**Methods:**

Using an exploratory approach, semi-structured focus groups were conducted separately with adolescents and (unrelated) parents. Participants were recruited from low and middle socio-economic areas in the Sydney metropolitan area and a regional area of New South Wales, Australia. Transcripts were analysed using thematic analysis for each of the four content areas (adolescent-ST, adolescent-SD consumption, parents' views on adolescents' ST and parents' views on adolescents' SD consumption).

**Results:**

Nine focus groups, with a total of 63 participants, were conducted. Broad themes spanned all groups: patterns of behavior; attitudes and concerns; adolescent-parent interactions; strategies for behavior change; and awareness of ST guidelines. While parents and adolescents described similar patterns of behaviour in relation to adolescents' SD consumption and ST, there were marked differences in their attitudes to these two behaviours which were also evident in the adolescent-parent interactions in the home that they described. Parents felt able to limit adolescents' access to SDs, but felt unable to control their adolescents' screen time.

**Conclusion:**

This study offers unique insights regarding topics rarely explored with parents or adolescents, yet which are part of everyday family life, are known to be linked to risk of weight gain, and are potentially amenable to change.

## Introduction

Overweight and weight-related behaviors developed during childhood and adolescence tend to track into adulthood, with significant long-term health implications [1-3]. Consumption of sugary drinks (SDs) and levels of sedentariness in this age group are of particular concern, given that there is probable and convincing evidence, respectively, that these behaviors are associated with increased risk of weight gain and the development of obesity [3-8]. In addition, there is evidence of a clustering of obesity-promoting behaviors, through the positive association between screen time (ST) and SD intake [9-12].

National and international guidelines consistently recommend that adolescents choose water as their main drink, keep their consumption of SDs to a minimum, and limit ST to less than 2 hours per day [13-15]. However, the evidence indicates that many adolescents fail to meet healthy eating guidelines and consume excessive volumes of SDs [5,16-18]. In a 2004 estimate in Australia 60% of boys and 40% of girls aged 12-16 years reported drinking more than 250 ml per day of soft drink [16]. Longitudinal research indicates that consumption of SDs tracks from adolescence into adulthood. [19] The majority of Australian adolescents also spend over 35 hours per week in screen time, and exceed recommendations to limit small screen recreation [20] The home environment contributes to SD and ST behavior patterns, through food availability, access to electronic media and television screens, and parental modeling [17,21,22]. SD and ST were thus selected as an appropriate focus of this study as they are specific behaviours related to weight status, potentially amenable to intervention within the home environment and particularly prevalent amongst adolescents [5].

While adolescents often experience a higher level of autonomy and independence compared with younger children [22], their parents and caregivers continue to play a key role in setting boundaries and supporting healthy lifestyle choices. However, little is known about how adolescents and their parents talk about weight and associated lifestyle behaviors, even though adolescent-parent relationships are a significant area of publication and research [23]. The nature of the interaction between parents and their adolescent dependents with regards to weight and associated behaviors is likely to impact on adolescents' perceptions and practices around these issues. Understanding adolescents' and parents' attitudes, concerns, and interactions about specific weight-related behaviours is fundamental to designing communication messages and obesity prevention interventions.

Therefore, the aim of this study was to explore adolescents' and parents' (of adolescents) perceptions, attitudes, and interactions in regards to adolescents' SD and ST within the home environment. The study had parallel research questions related to adolescents' and parents' perceptions:

◦ Adolescents:

(i) Are adolescents concerned about their SD consumption and recreational ST?

(ii) To what extent are these behaviors amenable to change? How could they be reduced?

◦ Parents of adolescents:

(i) Are parents concerned about their adolescents' SD consumption and ST?

(ii) To what extent do they see these behaviors as amenable to change? How could they be reduced?

◦ Both:

(i) What types of interactions occur at home regarding SD and ST? Are these topics discussed, and if so, how are they raised?

(ii) Are the views of parents and adolescents on SD and ST consistent or divergent?

## Methods

### Procedure

A qualitative study design using semi-structured focus groups was selected as an appropriate method of investigation for an initial exploration of perceptions and to gauge the breadth and strength of publicly expressed attitudes [24]. A market research company (MRC) was contracted to recruit participants and conduct the focus groups, in accordance with the study team's specifications. The study was approved by the Ethics Committees of The Children's Hospital at Westmead and The University of Sydney.

### Participants

Male and female adolescents in high school grades 8, 9 and 10 (i.e. 13-16 year olds) and (unrelated) parents or primary caregivers with at least one child in this age range were eligible to participate. The decision to recruit unrelated parents and adolescents was made to avoid contamination, as practical considerations meant that the researchers could not guarantee there could be simultaneous focus groups with related parents and adolescents.

Participants were recruited from residences in areas classified as low to middle socio-economic status (SES) in the Sydney metropolitan region and a regional centre of New South Wales, Australia. This study focused on adolescents and parents from low-middle socioeconomic groups because these groups tend to be at higher risk of the development of overweight [25]. SES was based on The Australian Bureau of Statistics Index of Relative Socio-economic Advantage/Disadvantage (IRSAD) [26] which is calculated for postal area codes. The IRSADs for each postcode in the Sydney Metropolitan region was ranked and divided into quintiles. People residing in postcodes in the bottom three quintiles (Q) were considered to be low (Q1), low-middle (Q2), and middle (Q3) SES and eligible for inclusion. The regional centre had an IRSAD that was equivalent to the metropolitan locations.

The MRC approached people listed in its database to participate in the study if they: had given consent to future contact; met the target group specifications; and had not participated in any form of market research in the past six months. Using their standard recruitment methods, the MRC contacted 457 parents by email and a further 33 by phone. In order to enhance recruitment in the regional centre, a subsequent email was sent to the remaining 187 non-eligible residents listed in the MRC's database with information about referring family and friends to the study. All participants received written information explaining the study. Informed consent by adolescents and by parents was a requirement for the study and participants were reimbursed for their travel expenses and time. Participants were required to speak fluent English in order to be able to participate fully in the focus group discussions.

### Data collection

Focus group discussion questions and prompts were developed to address the research questions, in a way that would promote interest and open discussion (see Table 1). Separate focus groups were conducted with parents and adolescents. Separate male and female adolescent groups were held as it was expected that adolescents would feel more comfortable discussing the study topic with peers of the same sex. Parent groups were arranged to comprise two combined mother-father groups and two separate mother and father groups. This arrangement was designed to ensure representation and active participation by fathers as well as mothers, recognizing that few studies actually seek out fathers.

**Table 1:** Discussion prompts

Parent focus groups
What do you think about the amount of time your child spends on screen-based leisure activities/their consumption of sugary drinks?
Tell me about any agreements, disagreements or rules you've had with your child about their use of screen-based leisure activities/consumption of sugary drinks?
If you wanted your child to reduce the amount of time they spend on screen-based leisure activities/the amount of sugary drinks they drink, what kind of strategies do you think would be effective?
Do you know whether there are any guidelines on the maximum amount of time adolescents should spend on screen-based leisure activities?

**Adolescent focus groups**

What do you think about the amount of time you spend watching TV/videos/DVDs or on computer games (not including time spent for educational purposes)?
How much do you care about your intake of sugary drinks?
Tell me about any agreements, disagreements or rules you've had with your parents about your use of screen-based leisure activities/consumption of sugary drinks?
What things would make it easier for people your age to drink less sugary drinks?

Each focus group session was conducted over 90-120 minutes and was held in a community-based meeting room. All sessions were digitally audio-recorded with participants' consent. A researcher from the study team attended and observed each session.

### Data Analysis

De-identified transcripts of the audio-recordings, typed verbatim, were provided by the MRC and checked for quality by a member of the research team. The transcripts were manually analyzed using thematic analysis. Three members of the research team independently read the transcripts and discussed the key ideas and common themes arising across the groups, and spanning the four content areas: adolescent-ST; adolescent-SD; parents' views on adolescents' ST; and parents' views on adolescents' SD. Following agreement on a draft coding structure, two members of the research team independently coded the data for each of the four content areas. The five broad themes comprised:

◦ Patterns of behavior

◦ Attitudes, beliefs and concerns;

◦ Adolescent-parent interactions;

◦ Strategies for behavior change;

◦ Awareness and perception of ST guidelines (the study did not specifically inquire regarding SD guidelines as there are no clear, precise guidelines).

A third member of the research team checked the results within each content area for consistency and minor revisions were made to the coding structure where appropriate. Consensus on the final coded data was reached in a straightforward manner and summaries of the findings were checked by members of the research team for accuracy. The five themes remained intact and it was agreed that they accurately captured the key information in the entire dataset, across all groups and content areas of interest, and in a way that addressed the overall research questions.

## Results

### Response rate

A total of 402 people responded to the initial study invitation, of whom 103 met the eligibility criteria. A final sample of 63 participated in a focus group (63% participation rate; 31 adolescents and 32 parents). A total of nine focus groups (five with adolescents; four with parents) were held in March 2008. Following completion of the nine focus groups, the primary and co-facilitator agreed that response saturation had been attained.

### Participant characteristics

Groups were primarily organized according to geographic location, with six in metropolitan Sydney and three in the regional centre (see Table 2). Adolescent participants in each group ranged in age between 13-16 years (Australian school Grades 8-10); 42% were female. The mean age of parent participants was 46 years for mothers and 43 years for fathers, and 63% were mothers. Approximately 40% of parents had finished high school or less as their highest education level, half had attained a tertiary certificate or diploma and just over 10% held a tertiary degree. Almost 20% of parent and 13% of adolescent participants were born outside Australia; and about 40% of adolescents were from families where at least one parent was born overseas. Almost one-third of adolescents and parents of adolescents lived in areas classified as low or low to middle SES, with the remainder of participants residing in areas ranked as middle SES.

**Table 2:** Focus group configuration

		Adolescents		Parents	
Group	Location	Male (n)	Female (n)	Fathers (n)	Mothers (n)
1	Metropolitan		8		
2	Metropolitan				8
3	Regional centre		5		
4	Regional centre	5			
5	Regional centre			2	6
6	Metropolitan	8			
7	Metropolitan			8	
8	Metropolitan	5			
9	Metropolitan			2	6

TOTAL		18	13	12	20

### Focus group discussion themes

The key findings and supporting quotations in relation to the major themes for each of the four content areas of interest were tabulated as a way of highlighting commonalities and differences between parents and adolescents, and between the behaviors of interest [see Additional file 1]. The range of comments in relation to these themes, across adolescents and parents and ST and SDs, are summarized below.

#### Behavior patterns

While carbonated soft drinks were largely discussed by both parents and adolescents as a 'treat' reserved for weekends, visitors or special occasions, other sugary drinks such as fruit juice and milk-based drinks were discussed as a daily component of adolescents' diets.

ST was overwhelmingly discussed as a part of daily life for adolescents. Parents also saw it as part of adolescents' daily life for communication and homework, and provided vivid and detailed descriptions of the times, occasions, purposes and preferences of their adolescents for using ST.

#### Attitudes, beliefs and concerns

For both ST and SD consumption, parents expressed noticeably more health-related concerns than adolescents.

Parents' concerns relating to SDs centered on the sugar and caffeine content, as well as acidity of various drinks, and resultant impacts on dental and general health. Interestingly, however, these concerns were not intensely or personally expressed, and not related to weight. Adolescents, on the other hand, expressed some mild health concerns relating to sugary drinks, including their contribution to weight.

Parents expressed a high degree of concern and frustration regarding their adolescent's ST. This was the foremost response to the topic in the parent groups. Parents were particularly concerned with the impact of excessive ST on social interaction and social skills, as well as competing with homework and learning. A few parents also raised concerns relating to ST and radiation exposure, repetitive strain injury and corruption of spelling. However, many of the parents accepted their adolescents' time spent in screen activities as inevitable and described themselves as 'giving up' and accepting ST as a feature of family life and society generally.

A common response from both parents and adolescents was that adolescents cannot be relied on to self-moderate their intake of SDs or use of ST. Further, parents frequently expressed a belief that they had little, or no, control over their adolescents' behaviors relating to ST. A common perception was that adolescents would ignore them, or go 'behind their back'. This was supported by comments from several adolescents who suggested that they would ignore or overcome any rules which were set with relative ease.

#### Adolescent-parent interactions

The nature of interactions between adolescents and parents varied depending on the behavior in question. Interactions regarding SDs were described as discussions where parents reminded the adolescent of what they should be drinking. In contrast, conversations focusing on ST were commonly described as being confrontational, with parents attempting to enforce rules. Both parents and adolescents noted that these discussions around ST frequently escalated into arguments and shouting.

Role modeling was identified as a relevant issue by some adolescents and parents in relation to SDs.

#### Strategies for behavior change

Common strategies identified by parents for promoting healthy behaviors in their adolescent children, in terms of SDs and ST, included controlling availability or access, and providing alternatives (particularly water). The adolescents perceived that this could work for SDs, but restrictions and enforced rules would be the best strategy for changing ST.

#### ST guidelines

There was limited awareness among both parents and adolescents about the guideline to limit small screen recreation time to less than 2 hours/day [13]. The question on this topic generated lots of different ideas about the rationale for a guideline. Mostly, parents thought that the 2 hour/day guideline wasn't feasible, was 'out of touch' and certainly would not be acceptable to adolescents. Some parents queried and derided the source or basis for the guideline and, in one group, there was an explicit dismissal of health guidelines generally. Some parents were concerned about how the guideline could be achieved in practice. Adolescents responded as parents anticipated, by rejecting the idea of a 2 hour/day limit as unrealistic and unacceptable.

### Participants' discussion style

While the major themes refer to discussion content, the analysis also identified differences between parents and adolescents in their style of discussion. Overall, parent focus groups involved more lively discussion than did the adolescent groups. Adolescents were slower to open up in discussion, and in some cases tended to express what might be taken as a publicly expected view, such as bragging about their ability to circumvent parental rules. In many ways, the issue of control was a present and overarching theme, with parents expressing the extent to which they can and cannot control their adolescent's behavior, and adolescents commenting on the extent they do and do not go along with parental control.

## Discussion

While the discussions on ST and SD consumption addressed similar themes (i.e. in terms of attitudes, interactions and behavior patterns), the nature of the attitudes and adolescent-parent interactions were quite different for each of these behaviors. Overall, neither parents nor adolescents expressed strong personal concern about adolescents' SD consumption, yet adolescents' ST elicited considerable concern amongst parents and was associated with family conflicts. Thus, these different behaviors had quite different emotional salience to parents and adolescents. Research with parents and adolescents has described a complex mix of factors, such as social expectations and marketing, parenting style and ability to say 'no', and communication taboos, that mediate perceptions and communications about weight [27]; some of which may be at play in this study.

Findings from this study that parents feel able to limit their children's access to SDs appear encouraging, given that previous research indicates that the home environment is critical in shaping children's dietary and activity behaviors [17,28,29], and that adolescents themselves recognize the importance of the home environment in influencing their behaviors [30]. However, there may be some discrepancy between parents' low level of personal concern and adolescents' actual consumption [16]. This mismatch, where 'treats' may in fact be consumed frequently, poses a barrier for change and needs to be anticipated in any communications or family interventions [27].

On the other hand, the extent to which parents felt unable to control their children's ST was concerning. For example, parents described how they had given up trying to limit ST, as a result of failed or negative interactions. Nevertheless, adolescents indicated that enforced rules were an appropriate control strategy. Comparing the adolescent and parent responses suggests that parental rules about ST are likely to lead to conflict, unless perhaps if rules are established when children are younger. No-one mentioned the use of TV time monitors or similar devices as a method for monitoring or enforcing rules.

While role modeling was mentioned in relation to SDs in both parent and adolescent groups, the limited emotional engagement with the topic suggests that its influence on adolescents continues to be under-estimated, especially by parents [27]. Research has highlighted that parents can be positive role models with their adolescent children in regard to weight-related behaviors [17,29,31]. There appears to be scope to promote the significance of role modeling and its applicability to influencing both SD consumption and ST to parents, through parent-targeted health communications and skill-based parenting interventions.

The low level of awareness of health recommendations and guidelines on recreational ST, and the dismissive response to the idea of guidelines, suggests that this guideline challenges public views and actual practices, which is consistent with findings from US qualitative research [32] and the context where Australian and North American family households have high access to electronic media. Recent data shows that 99% of Australian households with young people (8-17 years) have at least one television set, 98% have at least one computer or laptop, 97% a DVD player and 91% have an internet connection [21]. Clearly the health reasons for ST recreation guidelines, such as the association between recreational ST and fitness, is an important underpinning that should be featured in public health interventions and communications [33].

The typical roles that adolescents and parents play in relation to each other, with disagreements over everyday issues and struggles around control [23], were apparent in this study, even though the parents and adolescents were not related to each other and the group discussions were held separately. In this study parents frequently expressed issues in relation to their ability to control adolescents' behavior, and adolescents frequently asserted their independence from parental controls. While both positions correspond to an accepted public image, they also provide a reminder that any health communications and skill-based parenting interventions related to adolescents must always recognize and respond to this fundamental dynamic.

Overall, the study shows that there is scope to influence adolescents through their home environment and parental use of positive strategies, such as setting and applying defensible rules, limiting access and availability and role modeling. While an exploratory study, the findings suggest directions for health communication messages to parents and the value of structured parenting interventions, which typically focus on parents' understanding and acceptance of adolescent development as well as communication and listening skills [34].

While the study was generally successful in recruiting people from lower socioeconomic areas, there was difficulty in recruiting and retaining some participants from the most disadvantaged areas. The use of market research company recruitment methods may have limited contacts with people from low socio-economic backgrounds, although most eligible households are known to have internet access [21] and the purposive targeting of low SES areas meant that any potential bias was redressed. The study participants came from backgrounds that were fairly representative of the Australian population; although like many research studies, the views of the most disadvantaged, Aboriginal people or a wide range of people from culturally and linguistically diverse cultures are not comprehensively represented. Further research targeting these groups is required to investigate their particular environmental, cultural and material circumstances. While there is some possibility that participants were more health conscious than their peers, the recruitment information referred to 'lifestyle' rather than 'health' and health did not appear as a dominating theme. Overall, this study was successful in recruiting adolescents and parents from low and middle SES areas, with a particular strength in the participation of fathers, given that they are often under-represented.

The focus group design itself may have incurred some limitations, especially in relation to adolescents, who displayed a limited degree of 'conversational competency' in some cases [35] and tended to produce socially expected responses. However, it is important to understand and respond to these public views in designing communication and other interventions. Deeper understanding on specific issues, such as discrepancies between parents' level of concern and adolescents' actual behaviors, in relation to both SD and ST, would require additional research using a mix of methods, with related parent and adolescent participants.

Nevertheless, the study provides an initial exploration and offers insights regarding topics rarely explored in relation to adolescents, yet which frequently occur within the family and home environment, are known to be linked to risk of weight gain, and are discrete behaviors that are potentially amenable to change. The findings complement previous qualitative research with parents of younger children [27] and adolescents [30], as well as quantitative studies on these topics (for example [10,12,17,20]), thus providing guidance on angles and perspectives that can be used by policy makers, health practitioners and researchers to guide interventions and other health communications.

## Competing interests

The authors declare that they have no competing interests.

## Authors' contributions

Libby Hattersley (LHatt), Lesley King (LK), Vanessa Shrewsbury (VS), Louise Hardy (LH) and Louise Baur (LB) all contributed to the development of research questions and the design of the study, and the Ethics Committee submission. VS, LK and LHatt were involved in contracting the market research company and providing specifications and protocols for recruitment and conduct of groups. VS attended all focus groups and checked all transcripts. VS, LHatt, LK and Sarah Howlett (SH) undertook data coding and initial analyses; LH, LB, VS and SH were involved in checking the coding and analysis. All authors contributed to the interpretation of data and the writing of the manuscript. All authors read and approved the final manuscript.

## Supplementary Material

Additional file 1 **Key themes, concepts and quotes from adolescent and parent focus groups**. The table describes the key themes for parents and adolescents in relation to sugary drink and screen time, and provides illustrative quotes from focus group participants. Click here for file

## References

[B1] Lobstein T Baur L Uauy R Obesity in children and young people: a crisis in public health Obes Rev 2004 5 Suppl 1 4 104 10.1111/j.1467-789X.2004.00133.x 15096099

[B2] Wang LY Chyen D Lee S Lowry R The association between body mass index in adolescence and obesity in adulthood J Adolesc Health 2008 42 5 512 8 10.1016/j.jadohealth.2007.10.010 18407047

[B3] World Health Organization Diet, nutrition, and the prevention of chronic diseases:Report of a Joint WHO/FAO Expert Consultation 916 ed 2003 Geneva: World Health Organization 12768890

[B4] Crespo CJ Smit E Troiano RP Bartlett SJ Macera CA Andersen RE Television watching, energy intake, and obesity in US children: results from the third National Health and Nutrition Examination Survey, 1988-1994 Arch Pediatr Adolesc Med 2001 155 3 360 5 11231802 10.1001/archpedi.155.3.360

[B5] Gill TP Rangan AM Webb KL The weight of evidence suggests that soft drinks are a major issue in childhood and adolescent obesity Med J Aust 2006 184 6 263 4 16548828 10.5694/j.1326-5377.2006.tb00233.x

[B6] Ludwig DS Peterson KE Gortmaker SL Relation between consumption of sugar-sweetened drinks and childhood obesity: a prospective, observational analysis Lancet 2001 357 9255 505 8 10.1016/S0140-6736(00)04041-1 11229668

[B7] Tam CS Garnett SP Cowell CT Campbell K Cabrera G Baur LA Soft drink consumption and excess weight gain in Australian school students: results from the Nepean study Int J Obes (Lond) 2006 30 7 1091 3 10.1038/sj.ijo.0803328 16801946

[B8] Vartanian LR Schwartz MB Brownell KD Effects of soft drink consumption on nutrition and health: a systematic review and meta-analysis Am J Public Health 2007 97 4 667 75 10.2105/AJPH.2005.083782 17329656 PMC1829363

[B9] Kremers S Horst K van der Brug J Adolescent screen-viewing behaviour is associated with consumption of sugar-sweetened beverages: The role of habit strength and perceived parental norms Appetite 2007 48 3 345 50 10.1016/j.appet.2006.10.002 17126451

[B10] Salmon J Campbell KJ Crawford DA Television viewing habits associated with obesity risk factors: a survey of Melbourne schoolchildren Med J Aust 2006 184 2 64 7 16411870 10.5694/j.1326-5377.2006.tb00117.x

[B11] Van den BJ Van MJ Energy intake associated with television viewing in adolescents, a cross sectional study Appetite 2004 43 2 181 4 10.1016/j.appet.2004.04.007 15458804

[B12] Vereecken CA Todd J Roberts C Mulvihill C Maes L Television viewing behaviour and associations with food habits in different countries Public Health Nutrition 2006 9 02 244 50 10.1079/PHN2005847 16571179

[B13] The Australian College of Paediatrics Policy statement. Children's television J Paediatr Child Health 1994 30 1 6 8 10.1111/j.1440-1754.1994.tb00555.x 8148193

[B14] American Academy of Pediatrics Committee on Public Education. Children, Adolescents, and Television Pediatrics 2001 107 2 423 6 10.1542/peds.107.2.423 11158483

[B15] Smith A Kellet E Schmerlaib Y Australian Guide to Healthy Eating. Commonwealth of Australia 1998 Canberra: AGPS 0 642 27257 3

[B16] Booth ML Okely AD Denney-Wilson E Hardy LL Yang B Dobbins T NSW Schools Physical Activity and Nutrition Survey (SPANS) 2004: Full Report 2006 Sydney: NSW Department of Health ISBN 0 7347 3929 X

[B17] Campbell KJ Crawford DA Salmon J Carver A Garnett SP Baur LA Associations between the home food environment and obesity-promoting eating behaviors in adolescence Obesity (Silver Spring) 2007 15 3 719 30 10.1038/oby.2007.553 17372323

[B18] Hector D Rangan A Louie J Flood V Gill TP Soft drinks, weight status and health: a review 2009 Sydney: NSW Centre for Public Health Nutrition (now know as Cluster of Public health Nutrition, Prevention research Collaboration, University of Sydney)

[B19] Lien N Lytle LA Klepp KI Stability in consumption of fruit, vegetables, and sugary foods in a cohort from age 14 to age 21 Prev Med 2001 33 3 217 26 10.1006/pmed.2001.0874 11522162

[B20] Hardy LL Dobbins T Booth ML Denney-Wilson E Okely AD Sedentary behaviours among Australian adolescents Aust N Z J Public Health 2006 30 6 534 40 10.1111/j.1467-842X.2006.tb00782.x 17209269

[B21] ACMA Access to the internet, broadband and mobile phones in family households 2008 Australian Communications and Media Authority

[B22] Story M Neumark-Sztainer D French S Individual and environmental influences on adolescent eating behaviors J Am Diet Assoc 2002 102 3 Suppl S40 S51 10.1016/S0002-8223(02)90421-9 11902388

[B23] Smetana JG Campione-Barr N Metzger A Adolescent development in interpersonal and societal contexts Annual Review of Psychology 2006 57 1 255 84 10.1146/annurev.psych.57.102904.190124 16318596

[B24] Willis K Green J Daly J Williamson L Bandyopadhyay M Perils and possibilities: achieving best evidence from focus groups in public health research Aust N Z J Public Health 2009 33 2 131 6 10.1111/j.1753-6405.2009.00358.x 19413855

[B25] Shrewsbury V Wardle J Socioeconomic status and adiposity in childhood: a systematic review of cross-sectional studies 1990-2005 Obesity (Silver Spring) 2008 16 2 275 84 10.1038/oby.2007.35 18239633

[B26] Australian Bureau of Statistics Census of Population and Housing: Socio-economic indexes for areas (SEIFA) Australia 2006 2006 Canberra, Australian Bureau of Statistics. Information paper(Australian Bureau of Statistics) Ref Type: Internet Communication

[B27] Pagnini D King L Booth S Wilkenfeld R Booth M The weight of opinion on childhood obesity: recognizing complexity and supporting collaborative action Int J Pediatr Obes 2009 1 9 10.1080/17477160902763333 19922037

[B28] Grimm GC Harnack L Story M Factors associated with soft drink consumption in school-aged children J Am Diet Assoc 2004 104 8 1244 9 10.1016/j.jada.2004.05.206 15281041

[B29] Hardy LL Baur LA Garnett SP Crawford D Campbell KJ Shrewsbury VA Family and home correlates of television viewing in 12-13 year old adolescents: the Nepean Study Int J Behav Nutr Phys Act 2006 3 24 10.1186/1479-5868-3-24 16961929 PMC1594572

[B30] Booth ML Wilkenfeld RL Pagnini DL Booth SL King LA Perceptions of adolescents on overweight and obesity: the weight of opinion study J Paediatr Child Health 2008 44 5 248 52 10.1111/j.1440-1754.2007.01267.x 18194195

[B31] Horst K van der Oenema A Ferreira I Wendel-Vos W Giskes K van Lenthe F A systematic review of environmental correlates of obesity-related dietary behaviors in youth Health Educ Res 2007 22 2 203 26 10.1093/her/cyl069 16861362

[B32] Jordan AB Hersey JC McDivitt JA Heitzler CD Reducing children's television-viewing time: a qualitative study of parents and their children Pediatrics 2006 118 5 e1303 e1310 10.1542/peds.2006-0732 17079531

[B33] Hardy LL Dobbins TA Denney-Wilson E Okely AD Booth ML Sedentariness, small-screen recreation, and fitness in youth Am J Prev Med 2009 36 2 120 5 10.1016/j.amepre.2008.09.034 19135904

[B34] Henricson C Roker D Support for the parents of adolescents: a review J Adolesc 2000 23 6 763 83 10.1006/jado.2000.0358 11161338

[B35] Warr DJ "It was fun... but we don't usually talk about these things": Analyzing Sociable Interaction in Focus Groups Qualitative Inquiry 2005 11 2 200 25 10.1177/1077800404273412

