# Supplementary material for: Adolescent-parent interactions and attitudes around screen time and sugary drink consumption: a qualitative study
Source: Int J Behav Nutr Phys Act. 2009 Sep 9;6:61. doi: 10.1186/1479-5868-6-61 (PMC2747835; doi:10.1186/1479-5868-6-61)
Supplement: Additional file 1 — Key themes, concepts and quotes from adolescent and parent focus groups. The table describes the key themes for parents and adolescents in relation to sugary drink and screen time, and provides illustrative quotes from focus group participants. [file 1479-5868-6-61-S1.doc]

**Table 3. Key themes, concepts and quotes (italics) from adolescent and parent focus groups**

| **Theme** | **Sugary drinks** | | **Screen Time** | |
| --- | --- | --- | --- | --- |
|  | **Adolescents** | **Parents** | **Adolescents** | **Parents** |
| **Behavior patterns** | Soft drinks allowed as a treat or on special occasions; whereas juice and flavoured milk drinks consumed more regularly:  *“We’re only really allowed to have it [cordial or sweetened concentrate] when it’s an occasion or when someone comes over.” [Female]* | Soft drinks allowed as a treat for weekends or special occasions; whereas juice and flavoured milk drinks consumed more regularly:  *If we’ve got people over then the soft drink comes out. If there’s a whole bunch of kids over on the weekend then there’s cordial and maybe one bottle of soft drink.* | Part of daily life:  *“I guess screen activities are everywhere* *there is always a screen or something around you can watch something on.”* …*” [Female]*  Variation of uses, but mainly for leisure and communication:  *“When I come home that’s my ‘don’t talk to me, I’m on the internet time, wind-down time” [Female]* | Part of daily life:  *”Every day, all day.”*  Adolescents engage in SSR mostly for communication and homework:  *“As soon as my daughter comes home from school she’s on there chat-chat-chatting to her friends.”* |
| **Attitudes, beliefs and concerns** | Taste, caffeine and sugar content of soft drinks hold strong appeal:  *“[Water’s] just so boring. Like with all the new inventions of drinks and new mixes you can make and stuff, it just seems so bland.” [Female]*  Concerned largely about the present, some weight-related concerns:  *“Well if you drink too much – like I like playing sport, but if you drink too much fatty drinks or sugary drinks, you’ll get unfit and fat.” [Female]*  Concerns about artificially-sweetened drinks:  *"Coke is better than Diet Coke, because to make sure there is no sugar, it's got all chemical stuff in it, you will get sick and die.” [Male]*  Cannot be relied on to moderate own soft drink intake:  *“If I was allowed to drink Coke all day, I probably would." [Male]* | Concerns mostly about sugar, dental health, caffeine, acidity. Weight rarely mentioned:  *I know that the juices have got sugar in them even if they say they’re 100% juice but I do encourage – because I get it cheaper I encourage the boys to have it because they don’t eat much fresh fruit and so I figure it’s better – I’m trying to believe that they’re getting their other vitamins through the juice.”*  Concerned about the future  *“I don’t think my kids would buy the logic, they don’t think that far down the track. They think they just want that now.”*  Adolescents cannot be relied on to moderate intake:  *“You’re talking teenagers, it’s not a case of – well, I can’t say it’s only for Friday nights but we can sit and look at it in the fridge for the whole week, it’s not going to happen.”* | Few concerns:  *“No, I don’t care. We are kids.” [Male]*  Inevitability of ST as part of life:  *“I guess screen activities are everywhere, like they’re really hard to get away from…”*  Adolescents cannot be relied on to moderate screen-time:  *“I usually stretch my time on the computer and I get in trouble a lot, they won’t let me finish the game, we have arguments like that.” [Male]* | Wide range of concerns, particularly relating to social interaction:  *“It makes them anti-social. They don’t have-if you don’t push them away from it in some way shape or form, they are going to end up with very little social skills that they really need.”*  But ST inevitable part of home life and society in general:  *“Every child has got a Playstation or an X-Box or a Nintendo or a this or a that…”*  Adolescents cannot be relied on to moderate screen-time:  *“He’ll play it all day if he could. If I’m not there. If I’m there I’ll regulate it and say ‘get off’ but if I’m not there he’ll play X-box all day.”* |
| **Adolescent-parent interactions** | Low levels of family conflict. No rules set, or rules are disregarded:  *“Well they might say maybe stop drinking sugary drinks, not like you’ve got to stop, more like you should stop.”*  *“Yeah. I have the Red Bull and the V stuff because I’m not allowed to...that’s why I have them.” [Female]*  A need for parental role-modelling:  *“They can’t complain about me because they drink more than me” [Male]* | Low levels of family conflict. No rules set, or rules are disregarded:  *"It amuses me though they go to get a drink and you've got to just remind them ….'just have a drink of water' and so they will have it quite happily, but you have to remind them"*  *“Yes, when we go out for a meal she’ll always have a soft drink rather than juice. I always try and encourage her to have juice but she’ll always order a Coke.”*  A need for parental role-modelling:  *“My husband seems to think that if he doesn’t drink Coke after a meal he can’t digest his food. Of course the boys think if dad thinks that we’ll have some Coke too, Mum.”* | Often conflictual interactions:  *“They just keep repeating it like constantly and I’ll get annoyed and just get off.” [Female]*  Emphasis on non-compliance by adolescents:  *“Probably would be able to hack your way back through or something or my friend could” [Male]* | Often conflictual interactions:  *“I sat him down and started speaking slowly and nicely till it just got to the point where I was just screaming my head off saying ‘you don’t do this in our house….”*  Emphasis on non-compliance by adolescents:  *“Mine just ignore me…”* |
| **Strategies for behavior change** | Controlling drink availability – focus outside of home:  *“It’s because it’s always there, like in every shop there’s always something there that you might want, that you shouldn’t have but you want it so you get it anyway.” [Female]*  Having alternatives – focus on taste:  *“If they made it like soda water, if it had like artificial sugar stuff in it and it would taste good, then it would be better.” [Male]* | Controlling drink availability –focus on the home:  *“The majority of the time they’re home – I know they’re at school but it’s what you do the majority of the time that counts.”*  Having alternatives – focus on water:  *“Constant cold water in the fridge”* | Restricting time  *“I think like having a TV-free day or something” [Female]*  Limiting availability:  *“Turn off all in the internet access” [Male]*  Suggesting alternatives:  *“Get involved in physical activities, join an activity club” [Male]* | Restricting time the most common rule  *“I give XXX one to two hours on the computer and after that then he has to get off and do something else.”*  *“We made a rule at our place, no TVs in anyone’s bedroom…”*  Suggesting alternatives:  *“Encouraging them to do something positive with you, like going out to dinner or the beach or something they like doing.”* |
| **SSR Guidelines** | Not applicable | | Minimal awareness and dismissal of guideline as not being acceptable to adolescents:  *“There probably is but no one is going to follow them anyway.” [Male]* | Minimal awareness and dismissal of guideline as not being realistic:  *“No I haven’t heard that there’s such a guideline..”*  *“If I said that to my young lad he’d laugh at me”* |
